# Circulating Irisin Level and Thyroid Dysfunction: A Systematic Review and Meta-Analysis

**DOI:** 10.1155/2020/2182735

**Published:** 2020-10-28

**Authors:** Dan Shan, Li Zou, Xijiao Liu, Yitong Cai, Ruihong Dong, Yayi Hu

**Affiliations:** ^1^Department of Obstetrics and Gynecology, West China Second University Hospital, Sichuan University, Chengdu 610041, China; ^2^Key Laboratory of Birth Defects and Related Diseases of Women and Children, Sichuan University, Ministry of Education, Chengdu 610041, China; ^3^Department of Paediatric Surgery, West China Hospital of Sichuan University, Chengdu 610041, China; ^4^Department of Radiology, West China Hospital of Sichuan University, Chengdu 610041, China; ^5^West China School of Medicine, Sichuan University, Chengdu 610041, China

## Abstract

Both thyroid hormones and irisin have profound influences on the metabolism of the human body. Based on their similarities, several studies have been conducted to explore changes in irisin levels in patients with hypothyroidism and hyperthyroidism. This study was conducted in accordance with the PRISMA statement and the MOOSE reporting guideline. Based on a preregistered protocol (PROSPERO—CRD42019138430), a comprehensive search of eight databases was performed from inception to April 2020. Studies with original data collected from patients with thyroid dysfunction were included. Subgroup analysis was performed based on the different types of clinical manifestations and patient characteristics. The quality of each study and the presence of publication bias were assessed by the Newcastle-Ottawa score (NOS) and funnel plot with Egger's test, respectively. A total of 11 studies with 1210 participants were included. Ten studies were identified as high-quality studies. Pooled analysis indicated decreased irisin levels in patients with hypothyroidism (MD -10.37, 95% CI -17.81 to -2.93). Subgroup analysis revealed an even lower level of irisin in patients with clinical-type hypothyroidism (MD -17.03, 95% CI -30.58 to -3.49) and hypothyroidism caused by autoimmune disease (MD -19.38, 95% CI -36.50 to -2.26). No differences were found after achieving euthyroid status from levothyroxine treatment in patients with hypothyroidism compared with controls. No differences were found between patients with hyperthyroidism and controls. Correlation analyses revealed a possible negative correlation between irisin and TSH and positive correlations between irisin and both fT3 and fT4. Irisin was correlated with TSH receptor antibodies.

## 1. Introduction

Thyroid hormones have profound influences on the metabolic status of the human body; they can regulate both obligatory and facultative thermogenesis and have considerable effects on the cardiovascular system [1, 2]. Both hyperthyroidism and hypothyroidism are common, and the prevalence of hyperthyroidism is approximately 0.8% in Europe, 1.3% in the USA, and 1.1% in China [3–6]. The prevalence of spontaneous hypothyroidism is between 1% and 2% worldwide [7]. Patients with abnormal thyroid functions of either clinical-type or subclinical-type suffer from higher risks of cardiovascular disease, complications associated with altered lipid metabolism, and musculoskeletal system disorders [3, 8].

Irisin also plays an important role in regulating the metabolic status and influencing the function of the cardiovascular system [9, 10]. Irisin is the proteolytically cleaved form of fibronectin type III domain-containing protein 5 (FNDC5) [11]. Since the discovery of this protein, irisin has attracted widespread interest due to its broad physiopathological role in many metabolic diseases. Irisin has antioxidative, anti-inflammatory, and antiapoptotic effects, which indicates its potential as a therapeutic and diagnostic target for diabetes, obesity, nonalcoholic fatty liver disease, osteoporosis, and even cancer [10, 12, 13]. Irisin is involved in the expression of peroxisome proliferator-activated receptor- (PPAR-) gamma and uncoupling protein- (UCP-) 1, which are the hallmarks of thermogenesis [14]. Both triiodothyronine (T3) and irisin could increase the expression of UCP-1 levels, decrease lipid accumulation, and prevent DNA damage in human adipocytes [15]. However, based on a recently published research that used human subcutaneous white adipocytes, irisin and T3 might have different influences on adiponectin, PPAR-*γ*, and FNDC5 levels [16]. Animal studies have shown that the hypothalamic-pituitary-thyroid (HPT) axis function may be directly or indirectly related to irisin modulation, or vice versa [17–19].

Based on the similar physiological functions and possible connections of thyroid hormones and irisin, it is plausible to hypothesize that serum irisin levels may be altered in cases of thyroid dysfunction. Several studies have explored the function of irisin in patients with hypothyroidism and hyperthyroidism, but the evidence is conflicting. As a promising new diagnostic marker and a potential regulating hormone for metabolism, a systematic review summarizing the existing data regarding irisin levels in patients with thyroid dysfunction is needed.

The purposes of this study were to identify the difference in circulating irisin levels between participants with and without thyroid dysfunction and to explore the correlation between irisin levels with both thyroid hormones and antibodies.

## 2. Materials and Methods

We conducted this meta-analysis in accordance with the Meta-analysis Of Observational Studies in Epidemiology (MOOSE) guidelines for systematic reviews of observational studies [20]. In addition, our reporting items were in accordance with the Preferred Reporting Items for Systematic Reviews and Meta-Analyses (PRISMA) statement for reporting systematic reviews [21]. We followed a standard protocol, which was registered on PROSPERO of the Centre for Reviews and Dissemination (registration number: CRD42019138430). The method in this study was consistent with our previously published study [22].

### 2.1. Search Strategy and Selection Criteria

We performed a systematic search in four English electronic databases (PubMed, Embase, Web of Science, and Cochrane Central Register of Controlled Trials) and four Chinese electronic databases (the Chinese Biological Medical Literature database (CBM), the Chinese National Knowledge Infrastructure (CNKI) database, Chinese Medicine Premier (Wanfang database), and Chinese Journals Full-text Database (VIP)) without language restriction. The following search terms were used: “irisin”, “FDNC5”, “thyroid”, “hypothyroidism”, “hyperthyroidism”, “thyroid hormones”, and “thyroid antibodies”. The last search was performed in April 2020. If there were multiple publications based on a single study, the latest publication was used. We extracted supplemented data from the earlier versions. Reference lists within both the original literature and review articles were manually checked to identify eligible studies.

Studies were included if they met the following inclusion criteria: (1) studies reported original data (e.g., cross-sectional study, case-control study, and cohort study); (2) the primary outcome of interest was circulating irisin levels in patients with hypothyroidism or hyperthyroidism and healthy participants of euthyroid status; the secondary outcome was the correlation coefficients of irisin with thyroid hormones and antibodies; and (3) the diagnosis of hypothyroidism and hyperthyroidism was clearly defined [23, 24]. Studies were excluded if (1) the outcomes were not relevant or the diagnostic criteria of abnormal thyroid functions were not clearly defined, (2) case numbers were less than 5 in either arm, (3) the article was either a review article or a case report, and (4) the studies included participants who received treatment within three months or were currently receiving treatments, such as antithyroid treatments (ATDs), radioactive iodine, thyroidectomy, or levothyroxine therapy.

### 2.2. Data Extraction and Quality Assessment

After the initial evaluation, a specified data extraction form was used by two authors (DS and XL) to independently collect information. Any discrepancies were solved by conferring with other investigators. The following data were extracted: first author, study type, publication year, location of the studies, sample size, diagnostic criteria, related demographic characteristics, circulating irisin levels in different groups, and correlation coefficients of irisin with related thyroid hormones and antibodies. For the studies without available data in the paper, the first or corresponding author was contacted if necessary. For studies providing treatment to patients with thyroid dysfunction, the baseline irisin level was collected.

Two reviewers (LZ and XL) independently assessed the methodological qualities of the included studies in accordance with the Newcastle-Ottawa Quality Assessment Scale (NOS) independently. This scale evaluated observational studies based on three criteria: patient selection, comparability of study groups, and assessment of outcomes or exposure. The maximum score of the NOS is 9 points. Articles scoring more than 5 points were considered high-quality studies (with a low risk of bias). Articles that scored 1 or zero for selection of cases, scored zero for comparability of study groups, or scored zero for assessment of outcomes or exposure were considered low-quality studies (with a high risk of bias). Studies that scored in-between were categorized as having medium quality (with a moderate risk of bias). Any disagreements were resolved by discussion, with a third author involved in the decision.

### 2.3. Statistical Analysis

We performed meta-analyses using RevMan 5.3 (Ver 5.3. Copenhagen: The Nordic Cochrane Centre, The Cochrane Collaboration, 2014) and R (Ver 3.0.3. The R Project for Statistical Computing; http://www.r-project.org/). Additionally, the metafor package (Wolfgang Viechtbauer; http://www.metafor-project.org) was used. For data reported as medians, ranges, 95% confidence intervals, and *p* values, we calculated means and standard deviations [25, 26]. Because of the high likelihood of interstudy differences, a random effects model was first chosen. The *I*^2^ statistic was used to estimate heterogeneity among studies. Studies with an *I*^2^ value of <25%, 25%-50%, 50%-75%, or 75%-100% were considered to have no, low, moderate, or high heterogeneity, respectively [27]. If low heterogeneity was found, then a fixed effects model was used. The mean difference (MD) and the associated 95% confidence interval (CI) were calculated for circulating irisin in participants with and without hypothyroidism and hyperthyroidism. With respect to the correlation coefficients, statistical analyses were performed using the inverse of the variance method and Fisher's *z*-transformation. Subgroup analysis was performed based on the different types of clinical manifestations, the underlying disease, and study characteristics. Potential publication bias was evaluated by visual inspection of funnel plots as well as with Egger's regression asymmetry test. A two-tailed *p* value < 0.05 was considered statistically significant in all analyses.

## 3. Results

### 3.1. Characteristics of the Included Studies

**Figure 1:**
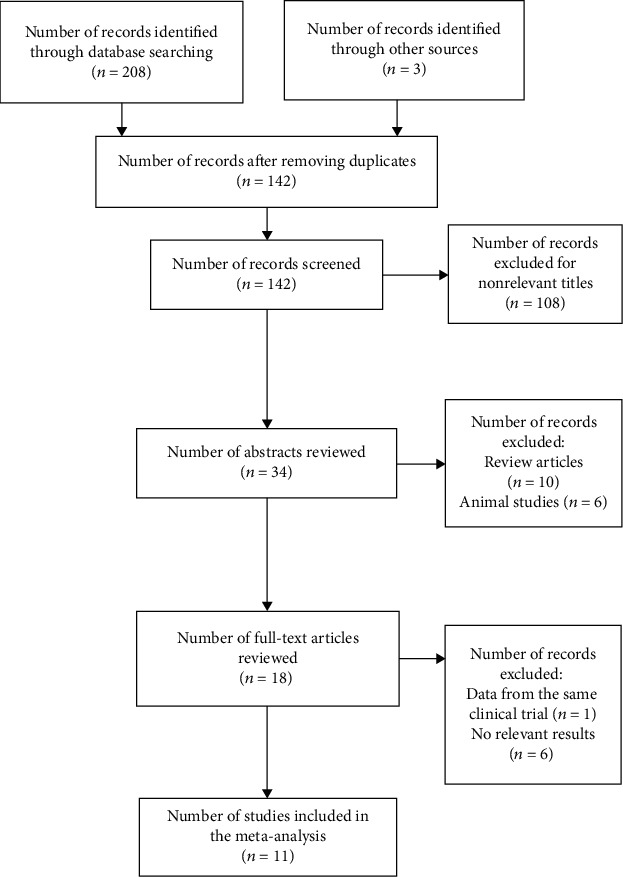
PRISMA flowchart showing the study selection process. PRISMA = Preferred Reporting Items for Systematic Reviews and Meta-Analyses.

The initial search identified 211 studies. Of these, 69 studies were excluded for duplicates. 108 studies were excluded after screening the titles. Another 23 studies were excluded after reading the abstract or full texts due to lack of relevant data or irrelevant study types (Figure 1).

A total of 11 studies with 1210 participants were included in this meta-analysis. All studies were single-centre observational studies. Five studies were based in Turkey [28–32], three studies were based in Poland [33–35], two studies were based in Greece [36, 37], and one study was based in China [38]. Six studies explored circulating irisin levels in patients with hypothyroidism [28, 30, 32, 35, 37, 38]; two of them included patients with subclinical hypothyroidism [32, 37]. Three studies explored circulating irisin levels in patients with hyperthyroidism [29, 31, 36]. Another two studies included patients with both hyper- and hypothyroidism [33, 34]. Autoimmune thyroid diseases (AITD) including Graves' disease, Hashimoto's thyroiditis, and other types were the most common reasons for thyroid dysfunction in these studies. In all included studies, the diagnostic criteria for hyper- and hypothyroidism were well defined and matched controls including age, sex ratio, and BMI were included. Some studies even used strict matching mechanisms involving blood pressure, eating habits, and waist-hip ratio [28, 36, 37]. Five studies treated patients with hypothyroidism with levothyroxine; irisin levels after treatment were reported [30, 32, 34, 37, 38]. The correlation coefficients of irisin with thyroid hormones and antibodies were reported in 9 studies [28–31, 33, 34, 36–38]. All blood samples were collected after fasting. Irisin was measured by commercial enzyme-linked immunosorbent assay (ELISA) kits from 4 different medical corporations. The main characteristics of the included studies are summarized in Table 1.

### 3.2. Irisin Levels in Patients with Hypothyroidism

**Figure 2:**
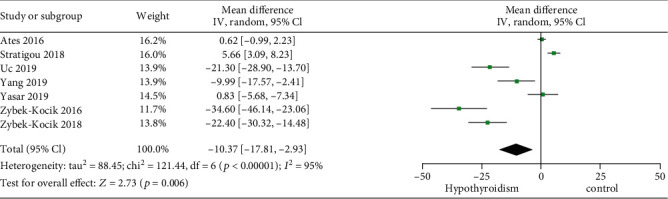
Forest plot for meta-analysis of seven studies on circulating irisin level in patients with hypothyroidism compared with controls. MD: mean difference; CI: confidence interval.

By pooling these studies using a random effects model, the results revealed that circulating irisin was lower in patients with hypothyroidism than in the controls (MD -10.37, 95% CI -17.81 to -2.93) (Figure 2).

### 3.3. Irisin Levels in Patients with Subclinical Hypothyroidism (SCH) and Patients with Clinical Hypothyroidism

Two studies reported circulating irisin levels in patients with SCH [32, 37], and there was no difference in irisin levels between patients with SCH and the control group (MD 4.21, 95% CI -0.13 to 8.55). However, in the comparison between patients with clinical-type hypothyroidism and controls, a significant decrease in the irisin level was revealed. Circulating irisin levels were significantly decreased in patients with clinical-type hypothyroidism (MD -17.03, 95% CI -30.58 to -3.49).

### 3.4. Irisin Levels in Patients with Hypothyroidism Caused by Autoimmune Disease

**Figure 3:**
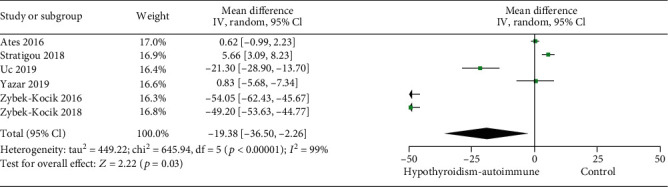
Forest plot for meta-analysis of six studies on circulating irisin level in patients with hypothyroidism of autoimmune disease compared with controls. MD: mean difference; CI: confidence interval.

AITD was the most common contributing reason for hypothyroidism in these studies. The pooling results showed that patients with autoimmune thyroid disease had a much lower irisin level than controls (MD -19.38, 95% CI -36.50 to -2.26) (Figure 3).

### 3.5. Irisin Levels in Patients with Hypothyroidism after Levothyroxine Treatment

Five studies reported irisin levels after levothyroxine treatment [30, 32, 34, 37, 38]. The treatment period ranged from 3 to 12 months, and most patients recovered to a euthyroid status. When comparing recovered patients after treatment with controls, no difference in circulating irisin levels was found (MD 1.48, 95% CI -2.69 to 5.65).

### 3.6. Irisin Levels in Patients with Hyperthyroidism

**Figure 4:**
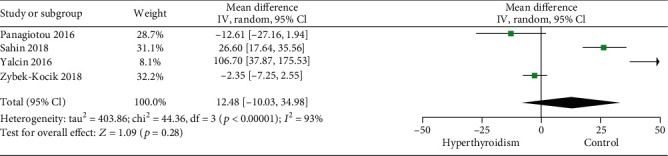
Forest plot for meta-analysis of four studies on circulating irisin level in patients with hyperthyroidism compared with controls. MD: mean difference; CI: confidence interval.

Four studies were eligible for the overall meta-analysis of irisin levels in patients with hyperthyroidism [29, 31, 34, 36]. Pooled analysis of these studies revealed no difference in irisin levels between patients with hyperthyroidism and the control group (MD 12.48, 95% CI -10.03 to 34.98) (Figure 4). No difference in circulating irisin levels was found in the comparison between patients diagnosed with Graves' disease and the control group (MD 24.11, 95% CI -5.14 to 53.37).

### 3.7. Correlation of Irisin with Thyroid Hormones

Correlation coefficients of irisin levels with thyroid hormones, including thyroid stimulating hormone (TSH), free triiodothyronine (fT3), and free thyroxine (fT4), of all participants were reported in seven studies [28–31, 33, 34, 37]. These pooling results from analyses including all participants did not reveal statistically significant associations between irisin levels and thyroid hormones (Table 2).

However, by excluding two studies [28, 37], we found a negative correlation of irisin with TSH (*r* -0.51, 95% CI -0.71 to -0.29) and positive correlations of irisin with fT3 and fT4 (*r* 0.35, 95% CI 0.09 to 0.60 in fT3; *r* 0.34, 95% CI 0.11 to 0.56 in fT4, respectively). Ates et al. compared irisin levels in patients with Hashimoto's thyroiditis with age- and BMI-matched controls. The ELISA kits used in their studies were different from those used in other studies. Stratigou et al. reported irisin levels in patients with subclinical hypothyroidism; however, six other studies were conducted in patients with clinical-type thyroid dysfunction. The differences in correlations between irisin levels and thyroid hormones might be contributed by differences in ELISA kits and differences in inclusion of participants.

Subgroup analyses were performed in patients with thyroid dysfunction and participants in the control group, respectively. In the patient group, correlation analyses of thyroid hormones and irisin showed no significant differences [28, 30, 36–38]. In patients with hypothyroidism [28, 30, 37, 38] and in patients with Hashimoto's thyroiditis [28, 30, 37], the pooling correlation coefficients of irisin with thyroid hormones had no statistical significance either. In the analysis of irisin levels with fT3 in patients with Hashimoto's thyroiditis, irisin was negatively correlated with fT3, but the correlation coefficient was small [30, 37]. In participants in the control group, irisin was negatively correlated with fT4 (*r* = −0.12, 95% CI -0.22 to -0.01) [28, 30, 31, 36, 37].

### 3.8. Correlation of Irisin and Thyroid Antibodies

Pooled correlation analysis between circulating irisin with antithyroid peroxidase antibodies (TPOAb) and antithyroglobulin antibodies (TGAb) showed no statistical significance [28–30, 34, 37] (*r* = 0.02, 95% CI -0.26 to 0.29 in TPOAb, and *r* = 0.02, 95% CI -0.32 to 0.36 in TGAb, respectively). However, analyzing three studies reporting irisin with TSH receptor antibodies (TRAb) indicated a positive correlation (*r* = 0.23, 95% CI 0.01 to 0.44) [29, 31, 34].

### 3.9. Assessment of Publication Bias and Sensitivity Analysis

**Figure 5:**
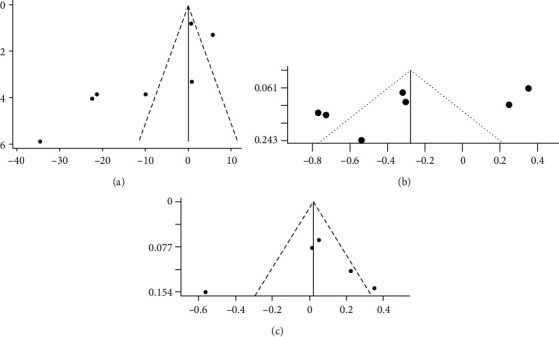
Funnel plots of included studies. (a) Irisin levels in patients with hypothyroidism compared with controls. (b) Correlation coefficient of irisin with thyroid stimulating hormone (TSH). (c) Correlation coefficient of irisin with antithyroid peroxidase antibodies (TPOAb).

Publication bias was assessed by funnel plots and Egger's test in circulating irisin levels with hypothyroidism (Figure 5(a)) and the correlations between irisin with TSH and TPOAb (Figures 5(b) and 5(c)). The funnel plot is symmetrically shaped, and no obvious publication bias was detected by Egger's test in the analysis of correlations between irisin and TSH and TPOAb (*p* = 0.09 and *p* = 0.70, respectively). However, possible publication bias was found in the analysis of irisin levels in patients with hypothyroidism (*p* = 0.04). After performing a sensitivity analysis by removing each study individually, the primary overall estimate was not substantially changed.

### 3.10. Assessment of the Quality of the Included Studies

The NOS scale was used to assess the quality of these studies (Table 3). Ten studies were of high quality and provided an adequate definition of cases confirmed by defined diagnostic criteria. They also provided clear records of participants' characteristics, comparability, and documentation of exposure and outcome. Only one study comparing the irisin level in patients with hyperthyroidism with hypothyroidism was categorized as having a moderate risk of bias with an NOS score of 5 [33]. As the baseline irisin levels were reported in all included participants and controls and no studies reported missing data, the response rates were judged to be 100%.

## 4. Discussion

**Figure 6:**
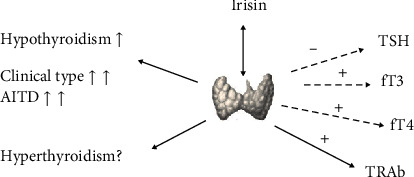
Schematic figure of the main findings. AITD: autoimmune thyroid disease; fT3: free triiodothyronine; fT4: free thyroxine; TSH: thyroid stimulating hormone; TRAb: TSH receptor antibodies.

This meta-analysis suggested that circulating irisin levels are lower in patients with hypothyroidism, especially in patients with clinical hypothyroidism and patients with autoimmune thyroid disease. Correlation analyses revealed possible connections of the irisin level with thyroid hormones. Pooling results from the analysis of the correlation between irisin and thyroid antibodies, including TPOAb and TGAb, were not statistically significant, but a possible association with TRAb was found (Figure 6). After levothyroxine treatment, the comparison of hypothyroid patients who recovered to a euthyroid status with healthy controls revealed no statistically significant difference. All of the included studies were conducted in the past 5 years. To the best of our knowledge, this is the first meta-analysis on circulating irisin levels in patients with thyroid dysfunction.

As important components in the regulation of metabolism and thermogenesis, both thyroid hormones and irisin have profound functions [3, 12, 39]. Animal studies have shown that irisin levels were changed in hyperthyroid and hypothyroid rat models [19, 40]. Our current analysis also confirmed the association of changing circulating irisin levels in patients with thyroid dysfunction, after application of good matching mechanisms such as age, sex ratio, body mass index, eating habits, and waist-hip ratio. The lower irisin level in patients with hypothyroidism implied possible energy metabolic disorders in these patients. In patients with clinical-type hypothyroidism and hypothyroidism caused by AITD, the abnormalities of energy metabolism might be even worse. These findings implied a possible link between hypothalamic-pituitary-thyroid (HPT) axis function and irisin levels. Despite the interplay of the HPT axis and irisin, the underlying reasons for the lower irisin level in hypothyroidism patients might also lie in the dysregulated lipid and glucose metabolic status of these patients. Hypothyroid conditions could lead to gradual deterioration of lipid metabolism and insulin sensitivity and might contribute to decreased irisin secretion [41]. The average body mass index (BMI) of patients with hypothyroidism in these included studies ranged from 24.7 to 30.2 kg/m^2^, which indicated the abnormalities in energy expenditure in these hypothyroid patients. However, in the analysis of patients with subclinical hypothyroidism, combining results yielded no statistically significant difference in irisin levels. In patients with hyperthyroidism, the pooling results implied no significant differences either.

A high level of heterogeneity was found in these analyses, and differences in clinical manifestations might be one contributing reason. Patients with clinical-type thyroid dysfunction might suffer from higher risks of metabolic disorder, as reflected by more prominent changes in irisin levels. Patients with the subclinical type and the healthy control group might not experience this dysregulation. Some studies conducted in patients without thyroid dysfunction reported no relationship between irisin and thyroid hormones [42, 43]. The duration of thyroid dysfunction might be another reason. In Zybek-Kocik's research, patients with long-lasting autoimmune thyroiditis and thyroidectomised subjects who were withdrawn from their levothyroxine treatment were included. They concluded that only long-lasting hypothyroidism results in significant decreases in irisin [35]. Four types of ELISA kits were used in these studies. Considering the large difference in irisin levels between studies, skepticism regarding the quality of commercial ELISA kits for irisin is justifiable. Most of our included studies used kits from the Phoenix company. Although previous studies measuring irisin levels using kits from different companies found no difference [44, 45], the results from correlation analyses indicated the possible influence of ELISA kits. The above reasons might contribute to the heterogeneities between studies. Beyond these possible contributing factors, differences in metabolic status, physical activities, and eating habits might also lead to heterogeneities between studies considering irisin's broad physiopathological functions and interactions with other myokines [9, 39].

The results from correlation analyses including all participants did not show associations between irisin and thyroid hormones. However, after excluding the two heterogeneous studies, we found possible associations of irisin levels with thyroid hormones in patients with clinical-type thyroid dysfunction. Despite the heterogeneities and the small *r* value, our findings indicated that a certain proportion of the thyroid hormone changes might be statistically explained by circulating irisin levels. Similar to our findings, analyses of irisin with bone mineral density and irisin with insulin resistance index also revealed associations [44, 46]. The possible influence of age, metabolic status, exercise, and personal medical histories could have potential effects. However, the correlation coefficients were small. The weak associations revealed by our research and others reflected a certain trend in irisin levels in one kind of population, but the complexity and variability of other influencing factors could not be ruled out. As the best serological marker for the diagnosis of autoimmune thyroiditis, serum TPOAb levels were elevated in patients with hypothyroidism in our included studies. However, we did not find an association between TPOAb and irisin. In patients with Graves' disease, TRAb plays a key role in the pathogenesis [47]. Our findings of the association between irisin and TRAb reflected this clinical course. Of note, irisin was found to have multiple physiopathological functions with many chronic diseases. Our previously published paper indicated the possible connection between irisin level and osteoporosis in senior patients [22]. In fact, since the discovery of irisin, several studies have been conducted to examine its correlation with many metabolic diseases, including polycystic ovary syndrome, diabetes, obesity, and nonalcoholic fatty liver disease [48–52]. As a micromolecule, irisin participates in many physiological processes. The potential of irisin as a therapeutic and diagnostic target for many metabolic diseases is still being explored.

Our current systematic review has several strengths. To our knowledge, this is the first meta-analysis assessing circulating irisin levels in patients with thyroid dysfunction. Most of the included studies were of good quality based on the NOS scoring system, which ensured the credibility of our results. All the studies used matched controls for age, and some studies used very detailed matching mechanisms. Well-defined diagnostic criteria and reliable library tests in the included studies guaranteed the reliability of our analyses to some extent. Additionally, a correlation coefficient analysis regarding both thyroid hormones and antibodies was performed, providing evidence of the association between irisin and thyroid function. However, some limitations should be noted. First, due to the limited number of included studies, only participants in Asian and European countries were included, which may not reflect the association of irisin and abnormal thyroid functions in other racial groups. Second, the number of included studies with eligible data was relatively small, especially in the analysis of irisin with thyroid antibodies. The connection between irisin and hyperthyroidism and subgroup analysis including patients with Graves' disease and Hashimoto's thyroiditis still need to be investigated. Third, a high level of heterogeneity was found in most of our analyses, possibly due to differences in participants' race, metabolic status, and physical activities between studies. However, most included studies did not evaluate these parameters, which should be further explored in future studies. Fourth, the possibility of underreporting of negative findings should not be ignored due to possible publication bias. The results should be interpreted with caution due to these limitations.

## 5. Conclusions

In conclusion, our meta-analysis revealed that circulating irisin levels were lower in patients with hypothyroidism. The correlation between irisin and thyroid hormones and antibodies indicated the possible interplay between them in regulating energy metabolism. Future studies should focus on including more patients of different racial groups and patients with subclinical types of thyroid dysfunctions. The connection between irisin and thyroid antibodies should be further explored. Studies with good control of all the confounding factors such as physical activity and metabolic complications are also needed.

## Figures and Tables

**Table 1:** Study characteristics.

First author	Year of publication	Location	Thyroid function	No. of cases	M/F	No. of control	M/F	Treatment	Irisin measurement	Outcomes investigated
Ateş	2016	Turkey	Hypothyroidism, Hashimoto's thyroiditis	37	4/33	37	1/36	N	Eastbiopharm (Hangzhou, Zhejiang, China)	Irisin level, correlation coefficients of irisin with thyroid hormones and thyroid antibodies
Panagiotou	2016	Greece	Subclinical hyperthyroidism, Graves' disease	16	1/15	80	8/72	N	Phoenix Pharmaceuticals (Burlingame, CA, USA)	Irisin level, correlation coefficients of irisin and thyroid hormones
Ruchala	2014	Poland	Hyperthyroidism, hypothyroidism	10 hyper; 10 hypo	1/9, 1/9	N	N	N	Phoenix Pharmaceuticals (Burlingame, CA, USA)	Correlation coefficients of irisin and thyroid hormones
Sahin	2018	Turkey	Hyperthyroidism, Graves' disease	25	10/15	24	6/18	N	Phoenix Pharmaceuticals (Burlingame, CA, USA)	Irisin level, correlation coefficients of irisin with thyroid hormones and thyroid antibodies
Stratigou	2018	Greek	SCH, Hashimoto's thyroiditis	120	48/72	120	48/72	Levothyroxine	Phoenix Pharmaceuticals (Burlingame, CA, USA)	Irisin level, correlation coefficients of irisin with thyroid hormones and thyroid antibodies
Uc	2019	Turkey	Hypothyroidism, Hashimoto's thyroiditis	26	2/24	19	5/14	Levothyroxine	Phoenix Pharmaceuticals (Burlingame, CA, USA)	Irisin level, correlation coefficients of irisin with thyroid hormones and thyroid antibodies
Yalcin	2016	Turkey	Hyperthyroidism, Graves' disease	41	26/15	44	31/13	N	Biovender (Brno, Czech Republic)	Irisin level, correlation coefficients of irisin with thyroid hormones and thyroid antibodies
Yang	2019	China	Hypothyroidism	77	14/63	78	12/66	Levothyroxine	Phoenix Pharmaceuticals (Burlingame, CA, USA)	Irisin level, correlation coefficients of irisin with thyroid hormones
Yasar	2019	Turkey	SCH, Hashimoto's thyroiditis	160	26/134	86	18/68	Levothyroxine	Sunred Biological Technology (Shanghai, China)	Irisin level
Zybek-Kocik	2016	Poland	Hypothyroidism, AITD, short term	24	2/22	12	1/11	N	Phoenix Pharmaceuticals (Burlingame, CA, USA)	Irisin level
Zybek-Kocik	2018	Poland	Hyperthyroidism, Graves' disease; hypothyroidism AITD, short term	55 hyper; 64 hypo	5/50, 7/57	45	6/39	Levothyroxine and antithyroid drugs	Phoenix Pharmaceuticals (Burlingame, CA, USA)	Irisin level, correlation coefficients of irisin with thyroid hormones and thyroid antibodies

Note: AITD: autoimmune thyroid disease; M/F: male/female; SCH: subclinical hypothyroidism.

**Table 2:** Pooled correlations between irisin with thyroid hormones.

Groups	No. of studies (no. of subjects)	*r*	95% CI	*I* ^2^	*p* value for heterogeneity	*p* value
*TSH*						
All participants	7 (677)	-0.27	(-0.55 to 0.06)	94.05%	<0.01	0.06
All participants^∗^	5 (363)	-0.51	(-0.71 to -0.29)	69.58%	0.01	<0.01
Patients with thyroid dysfunction	5 (276)	-0.09	(-0.36 to 0.18)	75.11%	<0.01	0.52
Patients with hypothyroidism	4 (260)	-0.05	(-0.35 to 0.26)	80.39%	<0.01	0.75
Patients with Hashimoto's thyroiditis	3 (183)	0.14	(-0.01 to 0.29)	0%	0.45	0.06
Control group	5 (334)	0.01	(-0.16 to 0.18)	50.78%	0.07	0.92
*fT3*						
All participants	5 (583)	0.23	(-0.05 to 0.49)	90.83%	<0.01	0.09
All participants^∗^	5 (363)	0.35	(0.09 to 0.60)	79.39%	<0.01	<0.01
Patients with thyroid dysfunction	4 (239)	0.00	(-0.25 to 0.25)	65.38%	0.02	0.99
Patients with hypothyroidism	3 (223)	-0.03	(-0.33 to 0.28)	75.91%	<0.01	0.86
Patients with Hashimoto's thyroiditis	2 (146)	-0.19	(-0.37 to -0.03)	0%	0.69	0.02
Control group	4 (297)	-0.05	(-0.16 to 0.07)	0%	0.60	0.42
*fT4*						
All participants	7 (677)	0.25	(-0.04 to 0.50)	92.25%	<0.01	0.13
All participants^∗^	5 (363)	0.34	(0.11 to 0.56)	73.85%	0.01	<0.01
Patients with thyroid dysfunction	5 (276)	0.10	(-0.17 to 0.37)	75.77%	<0.01	0.47
Patients with hypothyroidism	4 (260)	0.07	(-0.24 to 0.39)	82.06%	<0.01	0.65
Patients with Hashimoto's thyroiditis	3 (183)	-0.12	(-0.27 to 0.03)	0%	0.57	0.12
Control group	5 (334)	-0.12	(-0.22 to -0.01)	0%	0.72	0.04

Note: TSH: thyroid stimulating hormone; fT3: free triiodothyronine; fT4: free thyroxin; CI: confidence interval; *r*: correlation coefficient. ^∗^Two studies were excluded for including patients with subclinical hypothyroidism and application of different ELISA kits [28, 37].

**Table 3:** Newcastle-Ottawa score of the included studies.

	Selection	Comparability	Outcome/exposure	Total
Ateş 2016	∗∗	∗	∗∗∗	6
Panagiotou 2016	∗∗∗	∗∗	∗∗∗	8
Ruchala 2014	∗∗	∗	∗∗	5
Sahin 2018	∗∗	∗∗	∗∗∗	7
Stratigou 2018	∗∗∗	∗∗	∗∗∗	8
Uc 2019	∗∗	∗	∗∗∗	6
Yalcin 2016	∗∗	∗∗	∗∗∗	7
Yang 2019	∗∗	∗∗	∗∗∗	7
Yasar 2019	∗∗	∗	∗∗∗	6
Zybek-Kocik 2016	∗∗	∗∗	∗∗	6
Zybek-Kocik 2018	∗∗	∗∗	∗∗	6

Note: each “∗” present 1 point in the Newcastle-Ottawa score.
